# Usefulness of embryo evaluation via artificial intelligence–based image analysis

**DOI:** 10.20407/fmj.2025-016

**Published:** 2025-11-05

**Authors:** Takuma Kishida, Haruki Nishizawa

**Affiliations:** 1 Nishizawa Obstetrics and Gynecology Clinic, Iida, Nagano, Japan; 2 Department of Obstetrics and Gynecology, Fujita Health University, School of Medicine, Toyoake, Aichi, Japan

**Keywords:** Artificial intelligence, Embryo evaluation, Life Whisperer Viability

## Abstract

**Objective:**

Life Whisperer Viability (LW) is a software that evaluates embryos from a single image of blastocyst using artificial intelligence and is expected to improve the objectivity of embryo evaluation. In the present study, we retrospectively analyzed the clinical utility of LW.

**Methods:**

This study included 198 cycles in 135 patients who underwent frozen single embryo transfer. LW scores of transferred embryos were obtained from images taken approximately 116 h after fertilization. Based on LW scores, patients were divided into four groups (poor: 0–2.4; fair: 2.5–7.4; good: 7.5–8.9; excellent: 9.0–10), and rates of positive fetal heartbeat were compared among the groups. In addition, logistic regression analysis was performed to evaluate the association between LW score and the presence or absence of fetal heartbeat.

**Results:**

The rate of positive fetal heartbeat was significantly higher in the excellent group (64.3%, 36/56) than in the poor group (27.6%, 8/29) (*P*<0.05). Logistic regression analysis showed that the odds ratio for LW score was 1.150 (95% confidence interval: 1.040–1.280; *P*<0.05), indicating a significant association between LW score and presence or absence of fetal heartbeat.

**Conclusion:**

LW enables selection of embryos with a high potential for clinical pregnancy, demonstrating its clinical utility.

## Introduction

In assisted reproductive technology (ART), minimizing the financial and physical burdens on patients and achieving a live birth within a short period is essential. The method used to evaluate embryos for transfer is crucial in determining the time required to achieve a live birth.

Many ART facilities use the Gardner classification^1^ as the standard method for blastocyst evaluation. The Gardner classification independently evaluates blastocyst cavity expansion and hatching status on a six-point scale (1–6). It evaluates the morphology of the inner cell mass and trophectoderm on a three-point scale (A–C), with results represented by scores such as 4AB. The evaluation method using the Gardner classification is simple, and high-grade embryos have a higher pregnancy rate than low-grade embryos, indicating its high usefulness as an assessment tool.^2^ However, a limitation of this method is the subjectivity involved in scoring, resulting in interobserver variability. Another limitation is the diversity of evaluation results, making it difficult to decide which embryos have the highest pregnancy potential. For example, it can be challenging to prioritize between embryos graded 4BB and 3AA for transfer.

Life Whisperer Viability (LW), developed by Presagen (Australia), is a web-based embryo evaluation system that uses artificial intelligence to assess embryos from a single blastocyst image.^3^ The LW evaluation (LW score) is a quantitative score ranging from 0 to 10, with higher scores indicating a greater likelihood of positive fetal heartbeat following embryo transfer. This system is expected to provide highly objective evaluations, and by converting results into quantitative data, it may facilitate prioritization of embryos for transfer.

In the present study, to assess the clinical utility of LW, we retrospectively evaluated embryos previously transferred at our hospital using LW and analyzed the association between LW score and the presence or absence of fetal heartbeat.

## Methods

We included a total of 198 cycles in 135 patients who underwent frozen-thawed single blastocyst transfer at our hospital between April 2020 and August 2023. This opt-out study was approved by our institutional ethics committee. There is no conflict of interest associated with this study.

Ovarian stimulation was performed using the GnRH antagonist protocol, clomiphene+hMG, or progestin-primed ovarian stimulation, selected according to the treatment plan for each patient, and egg retrieval was performed under transvaginal ultrasound 35 h after human chorionic gonadotropin (hCG) administration. Retrieved oocytes were precultured for 3–4 h and inseminated by conventional in vitro fertilization (IVF) or intracytoplasmic sperm injection (ICSI). Normal fertilization was determined by confirming the presence of two pronuclei approximately 19 h after insemination. Embryos were cultured in single medium, and frozen/thawed using the Cryotop method.^4^ Frozen-thawed embryo transfer was performed during hormone replacement cycles using estradiol and progesterone. Expanded blastocysts cryopreserved on day 5 after fertilization were used for embryo transfer. Pregnancy was determined 2 weeks after embryo transfer; subsequently, pregnancy progress was monitored every other week. Patients in whom fetal heartbeat was confirmed were considered patients with positive fetal heartbeat.

Evaluation by LW was performed using embryo images taken approximately 116 h after insemination. Based on the obtained LW scores, the patients were divided into four groups (poor: 0–2.4; fair: 2.5–7.4; good: 7.5–8.9; excellent: 9.0–10) with reference to previous reports.^3^ In addition, because the developer of LW recommends using images in which the inner cell mass is in focus for embryo evaluation, images in which it was clearly out of focus were excluded from this study.

In Analysis 1, rate of positive fetal heartbeat was compared among the four groups. Fisher’s exact test, one-way analysis of variance, Kruskal–Wallis test, and Bonferroni correction were used for statistical analyses. In Analysis 2, logistic regression analysis was performed with the presence or absence of fetal heartbeat as the dependent variable, the LW score as the independent variable, and the maternal age and number of previous transfers as confounding factors, to examine the association between the LW score and the presence or absence of fetal heartbeat.

## Results

### Analysis 1

The patient characteristics for each group are presented in Table 1. There were no significant differences in maternal age and number of previous transfers between the groups.

The rates of positive fetal heartbeat were 27.6% (8/29) in the poor group, 42.7% (35/82) in the fair group, 41.9% (13/31) in the good group, and 64.3% (36/56) in the excellent group. The rate in the excellent group was significantly higher than that in the poor group (*P*<0.05) (Table 2).

### Analysis 2

Logistic regression analysis was performed with the presence or absence of fetal heartbeat as the dependent variable, LW score as the independent variable, and maternal age and number of previous transfers as confounding factors, and the odds ratio for LW score was 1.150 (95% confidence interval: 1.040–1.280; *P*<0.05), indicating that LW score was significantly associated with the presence or absence of fetal heartbeat (Table 3).

## Discussion

To assess the usefulness of embryo evaluation using LW, this study analyzed data from embryo transfers previously performed at our hospital. In Analysis 1, when the patients were divided into four groups based on LW scores, a significant difference was observed in the rate of positive fetal heartbeat between the group with the lowest LW score (poor) and that with the highest LW score (excellent). In Analysis 2, logistic regression was performed with adjustments for maternal age and the number of previous embryo transfers, which are factors considered to influence the presence or absence of a fetal heartbeat, as confounders. The analysis revealed that the LW score significantly affects fetal heartbeat presence. The results of this study indicate that the use of LW as an evaluation method for selecting blastocysts for transfer has clinical utility. In addition, when the embryos used in this study were evaluated using the Gardner classification, the rate of positive fetal heartbeat in embryos with the best grades (4AA, 5AA, and 6AA) was 55.4% (41/74, unpublished data), which tended to be lower than that of the excellent group (64.3%), indicating that LW evaluation can select embryos with higher fertility compared with the evaluation by the Gardner classification.

**Figure 1:**
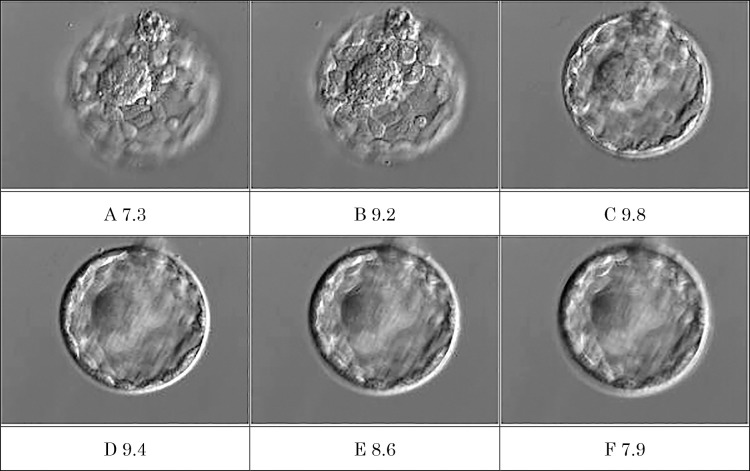
Changes in LW scores for the same embryo photographed at different focal depths The images were taken by gradually changing the focus from A to F. Embryo diameter was 180 μm.

Embryo evaluation based on the Gardner classification classifies embryos into six stages using blastocyst cavity expansion and hatching status as indicators. In addition, after stage 3, the inner cell mass and trophectoderm are each evaluated on a scale of three stages. Because this type of evaluation lacks clear boundaries and relies on evaluator subjectivity, assessments can vary between evaluators.^5^ To minimize interobserver differences in embryo assessment, it has been reported effective to assign all evaluations to a single evaluator, to reach consensus among multiple evaluators,^6^ or to simplify the criteria and reduce the number of evaluation patterns.^7^ However, achieving consistent evaluations remains challenging. In addition, the Gardner classification includes a total of 38 embryo evaluation patterns, making it difficult to immediately identify which embryos have high pregnancy potential. In contrast, LW offers high reproducibility because of its objective evaluation method, consistently producing the same results regardless of how many times the same image is assessed. Furthermore, because LW provides a quantitative score ranging from 0 to 10, it can help clearly identify embryos with higher pregnancy potential, thereby facilitating communication and decision-making between physicians and patients regarding embryo selection. In the laboratory, reducing the time required for embryo evaluation also shortens the duration that embryos are exposed outside the incubator, which may contribute to improved culture quality. Furthermore, it is expected to shorten training on embryo evaluation and eliminate problems arising from differences in evaluations by different evaluators. However, a limitation of LW is that the LW scores of the same embryo differ when images are taken with different focuses (Figure 1). The developer recommends that images focused on the inner cell mass be used for embryo evaluation and care should be taken when taking these images.

In addition to LW, iDA Score (Vitrolife) is another embryo evaluation method that uses artificial intelligence. iDA Score is an embryo evaluation system installed in a time-lapse incubator that automatically analyzes and scores the morphology and developmental dynamics of embryos in culture.^8^ The usefulness of iDA Score has been reported, in which high-grade embryos have a lower miscarriage rate and higher live birth rate than low-grade embryos.^9^ The iDA Score differs from LW in that it incorporates developmental dynamics observed during culture for a more detailed evaluation. However, its drawbacks include the need for a costly time-lapse incubator and the requirement that embryos be continuously cultured in this system for evaluation to be possible. In contrast, LW is more convenient because evaluation can be made with only one image of the blastocyst.

In 2017, the European Society of Human Reproduction and Embryology established key performance indicators (KPIs) for ART outcomes,^10^ and IVF laboratories should use these indicators to regularly review and improve the culture environment by comparing them with the facility’s own performance. In addition, studies and conference presentations often compare outcomes using “good blastocysts” as an indicator. However, the criteria for what constitutes a “good” embryo vary by facility and evaluator, resulting in data that lack objectivity and standardization. In this context, an objective embryo evaluation system such as LW would be useful for establishing a common indicator among facilities. Potential applications of this method include comparing the development rates of high-grade embryos (those with an LW score ≥9.0) and establishing the pregnancy rate of transferred high-grade embryos as a KPI. In the present study, the fetal heartbeat positivity rate in the excellent group (LW score ≥9.0) was 64.3%. Similarly, in the report by Diakiw et al.,^3^ the rate for the same group was 64.6%, indicating nearly identical results. This indicates that the quality of embryo transfer outcomes, as measured by the fetal heartbeat positivity rate, is comparable between the facility of Diakiw et al. and our own.

The results of the present study indicate that using LW makes it possible to select embryos with a higher likelihood of positive fetal heartbeat after embryo transfer, demonstrating the clinical utility of embryo evaluation using artificial intelligence–based LW. This allows for highly objective embryo evaluation and is expected to enhance both patient understanding and culture quality. However, currently, evaluation can only be performed on blastocysts on day 5 of culture, and blastocysts on day 6 of culture were not included, which is an area for improvement. In the future, improvements in the accuracy of LW as an embryo evaluation system and expansions in its analysis patterns are anticipated. On the user side, it will be important to closely examine the relationships between maternal age, embryo culture duration, and LW scores.

## Figures and Tables

**Table 1:** Patient characteristics of each group

Group	Poor	Fair	Good	Excellent	Total
Range of LW^#^ score	0.0–2.4	2.5–7.4	7.5–8.9	9.0–10.0	
No. of cycles	29	82	31	56	198
Age (Mean±SD)	37.1±4.2	36.2±4.6	34.3±4.5	35.1±4.8	35.7±4.6
Median (IQR) number of transferred	1 (0–1)	0.5 (0–2)	1 (0–1)	0 (0–1)	0 (0–1)
Causes of infertility					
Tubal factor	0 (0%)	6 (7.3%)	1 (3.2%)	3 (5.4%)	10 (5.1%)
Endometriosis	3 (10.3%)	8 (9.8%)	1 (3.2%)	3 (5.4%)	15 (7.6%)
Male factor	9 (31.0%)	17 (20.7%)	5 (16.1%)	8 (14.3%)	39 (19.7%)
Ovulation	1 (3.4%)	3 (3.7%)	5 (16.1%)	4 (7.1%)	13 (6.6%)
Low Anti-Mullerian Hormone levels	1 (3.4%)	1 (1.2%)	0 (0%)	0 (0%)	2 (1.0%)
Age factor	2 (6.9%)	4 (4.9%)	3 (9.7%)	7 (12.5%)	16 (8.1%)
Multiple	5 (17.2%)	6 (7.3%)	3 (9.7%)	5 (8.9%)	19 (9.6%)
Unknown	8 (27.6%)	37 (45.1%)	13 (41.9%)	26 (46.4%)	84 (42.4%)

^#^ LW: Life Whisperer Viability

**Table 2:** Comparison of fetal heartbeat positivity rates among four LW score–based groups

Groups	Poor	Fair	Good	Excellent
No. of cycles	29	82	31	56
No. (%) of positive fetal heart beat	8 (27.6%)^a^	35 (42.7%)	13 (41.9%)	36 (64.3%)^b^

^a,b^ Significant difference between these values (*P*<0.05)

**Table 3:** Effect of LW score on the presence or absence of fetal heartbeat

	Odds ratio	95% confidence interval	*P* value
LW^#^ score	1.150	1.040–1.28	0.009
Age	0.915	0.855–0.98	0.012
No. of transferred	0.831	0.630–1.10	0.189

^#^ LW: Life Whisperer Viability

## Supplementary Material

PDF-Japanese

## References

[1] Gardner DK, Lane M, Stevens J, Schlenker T, Schoolcraft WB. Blastocyst score affects implantation and pregnancy outcome: towards a single blastocyst transfer. Fertil Steril 2000; 73: 1155–1158. 10856474 10.1016/s0015-0282(00)00518-5

[2] Zhao YY, Yu Y, Zhang XW. Overall blastocyst quality, trophectoderm grade, and inner cell mass grade predict pregnancy outcome in euploid blastocyst transfer cycles. Chin Med J 2018; 131: 1261–1267. 29786036 10.4103/0366-6999.232808 PMC5987494

[3] Diakiw SM, Hall JMM, VerMilyea M, et al. An artificial intelligence model correlated with morphological and genetic features of blastocyst quality improves ranking of viable embryos. Reprod Biomed Online 2022; 45: 1105–1117. 36117079 10.1016/j.rbmo.2022.07.018

[4] Kuwayama M, Vajta G, Kato O, Leibo SP. Highly efficient vitrification method for cryopreservation of human oocytes. Reprod Biomed Online 2005; 11: 300–308. 16176668 10.1016/s1472-6483(10)60837-1

[5] Storr A, Venetis CA, Cooke S, Kilani S, Ledger W. Inter-observer and intra-observer agreement between embryologists during selection of a single Day 5 embryo for transfer: a multicenter study. Hum Reprod 2017; 32: 307–314. 28031323 10.1093/humrep/dew330

[6] Baxter Bendus AE, Mayer JF, Shipley SK, Catherino WH. Interobserver and intraobserver variation in day 3 embryo grading. Fertil Steril 2006; 86: 1608–1615. 17074349 10.1016/j.fertnstert.2006.05.037

[7] Richardson A, Brearley S, Ahitan S, Chamberlain S, Davey T, Zujovic L, Hopkisson J, Campbell B, Raine-Fenning N. A clinically useful simplified blastocyst grading system. Reprod Biomed Online 2015; 31: 523–530. 26283016 10.1016/j.rbmo.2015.06.017

[8] Berntsen J, Rimestad J, Lassen JT, Tran D, Kragh MF. Robust and generalizable embryo selection based on artificial intelligence and time-lapse image sequences. PLoS One 2022; 17: e0262661. 35108306 10.1371/journal.pone.0262661 PMC8809568

[9] Ueno S, Berntsen J, Ito M, Okimura T, Kato K. Correlation between an annotation-free embryo scoring system based on deep learning and live birth/neonatal outcomes after single vitrified-warmed blastocyst transfer: a single-centre, large-cohort retrospective study. J Assist Reprod Genet 2022; 39: 2089–2099. 35881272 10.1007/s10815-022-02562-5 PMC9475010

[10] ESHRE Special Interest Group of Embryology and Alpha Scientists in Reproductive Medicine. The Vienna consensus: report of an expert meeting on the development of ART laboratory performance indicators. Reprod Biomed Online 2017; 35: 494–510. 28784335 10.1016/j.rbmo.2017.06.015

