# Supplementary material for: Usefulness of embryo evaluation via artificial intelligence–based image analysis
Source: Fujita Med J. 2025 Nov 5;12(1):29–32. doi: 10.20407/fmj.2025-016 (PMC12865280; doi:10.20407/fmj.2025-016)
Supplement: Supplementary file 1 — PDF-Japanese [file fmj-12-029-s001.pdf]

Original Article

タイトル：人工知能を活用した画像解析による胚評価の有用性

著者：岸田拓磨 [1)]、西澤春紀 [1,2)]

Takuma Kishida, Master of Assisted Reproductive Technology, Haruki Nishizawa, MD, PhD

所属：

[1)] 西澤産婦人科クリニック Nishizawa Obstetrics and Gynecology Clinic, Iida, Nagano, Japan

[2)] 藤田医科大学産婦人科 Department of Obstetrics and Gynecology, Fujita Health University, School of Medicine, Toyoake, Aichi, Japan

ランニングタイトル：人工知能による胚評価

Corresponding author: Takuma Kishida, Master of Assisted Reproductive Technology

Address: 4-5, Honmachi, Iida, Nagano 395-0044, Japan

0265-24-3800

nishizawa.ivf@gmail.com

【抄録】

目的：Life Whisperer Viability（以後 LW）は胚盤胞の画像 1 枚から人工知能によって胚を評価するソフトウェアであり、胚評価の客観性向上が期待される。今回、LW の有用性を後方視的に検討した。方法：凍結単一胚移植を行った 135 症例 198 周期を対象とした。移植胚は受精から約 116 時間後に撮影した胚画像を用いて LW 値を得た。LW 値をもとに 4 群（poor；0 ～ 2.4、fair；2.5 ～ 7.4、good；7.5 ～ 8.9、excellent；9.0 ～ 10）に分け、各群の胎児心拍陽性率を比較した。また、ロジスティック回帰分析を行い、LW 値と胎児心拍の有無との関連性を検討した。結果：Excellent の胎児心拍陽性率は 64.3% (36/56) であり、poor の 27.6% (8/29) よりも有意に高い結果となった（$P < 0.05$）。ロジスティック回帰分析の結果、LW 値のオッズ比は 1.150 (95%信頼区間 1.040-1.28：$P<0.05$）であり、LW 値は胎児心拍の有無との関連が認められた。結論：LW によって臨床妊娠の可能性が高い胚を選択でき、臨床における有用性が明らかとなった。

【キーワード】

人工知能、胚評価、Life Whisperer Viability

【序論】

生殖補助医療（以後 ART）において、患者の経済的・身体的負担を最小限に留めつつ短期間で生児を獲得することが重要であり、移植胚を選択する際に用いる胚評価法は生児獲得までの期間を左右する重要な因子となる。

多くの ART 施設では、胚盤胞の評価法として Gardner 分類[1] を使用している。Gardner 分類は胚盤胞腔の拡がりと孵化の状態を 1 から 6 の 6 段階、内部細胞塊および栄養外胚葉の形態をそれぞれ A から C の 3 段階で独立して評価し、4AB のように表記する。Gardner 分類を用いた評価法は容易であり、高グレード胚の妊娠率は低グレード胚よりも高く、評価としての有用性が高い[2]。一方で、評価を付ける際に評価者の主観が入りやすく評価に個人差が生じやすいことが欠点と言える。また、評価結果が多様であり、どの胚が妊娠率の高い胚か判断が難しいことも欠点である。例えばグレード 4BB と 3AA の胚があった場合にどちらを優先して移植するべきか判断に迷う。

Presagen 社（豪州）が開発した Life Whisperer Viability (以後 LW）は、1 枚の胚盤胞の画像から人工知能に基づいて胚を評価するウェブ上の胚評価システムである[3]。LW による評価（以後 LW 値）は 0 から 10 の数値で算出され、10 に近いほど胚移植後の胎児心拍が陽性になる可能性が高い胚であると判定される。これにより、客観性が高い評価が期待され、さらには評価結果が量的データになることで移植する胚の優先順位が決めやすくなることも期待される。

今回、LW を用いた胚評価が臨床上有用であるか検討するため、当院で過去に移植した胚を LW で評価を行い胎児心拍の有無との関連性を分析した。

【方法】

2020 年 4 月から 2023 年 8 月までに当院で凍結融解単一胚盤胞移植を行った 135 症例 198 周期を対象とした。なお、本研究は当院倫理委員会の承認のもと、オプトアウトを行い実施した。なお、本研究に関する利益相反は無い。

卵巣刺激は GnRH アンタゴニスト法やクロミフェン＋hMG 法、Progestin-primed Ovarian Stimulation (PPOS) 法を各症例の治療方針に合わせて選択して行い、hCG 投与から 35 時間後

に経腟超音波下で採卵を行った。回収した卵子は 3〜4 時間の前培養後に conventional-IVF もしくは ICSI によって媒精を行った。媒精から約 19 時間後に 2 前核の確認を持って正常受精と判定した。胚培養は single medium を用い、胚の凍結と融解は cryotop 法[4] で行った。凍結融解胚移植はエストラジオール製剤とプロゲステロン製剤を用いたホルモン補充周期に行った。移植胚には受精 5 日目に凍結保存した拡張胚盤胞を用いた。胚移植から 2 週間後に妊娠判定を行い、妊娠判定以降は 1 週間おきに妊娠経過を確認し、胎児心拍が確認できた症例を胎児心拍陽性とした。

LW による評価は、媒精から概ね 116 時間後に撮影した胚画像を用いて行った。得られた LW 値をもとに既報[3] を参考にして 4 群（poor；0〜2.4、fair；2.5〜7.4、good；7.5〜8.9、excellent；9.0〜10）に分けた。なお、LW の開発元は胚評価に当たって内細胞塊にピントが合っている画像を用いることを推奨しているため、明らかにピントがずれている画像は今回の研究対象から除外した。

検討 1 では 4 群間の胎児心拍陽性率を比較した。統計解析には Fisher の正確確率検定や一元配置分散分析、Kruskal-Wallis 検定、Bonferroni 補正を用いた。検討 2 では、目的変数を胎児心拍の有無、説明変数を LW 値と妻年齢、既往移植回数としてロジスティック回帰分析を行い、LW 値と胎児心拍の有無との関連性を検討した。

【結果】

検討 1

各群の患者背景を表 1 に示した。妻年齢と既往移植回数について各群間において有意な差は認められなかった。

各群の胎児心拍陽性率は、poor が 27.6% (8/29)、fair が 42.7% (35/82)、good が 41.9% (13/31)、excellent が 64.3% (36/56) であり、excellent は poor よりも有意に高い結果となった（$P < 0.05$）(表 2)。

検討 2

目的変数を胎児心拍の有無とし、説明変数に LW 値の他、交絡因子として妻年齢と既往移植回数を加えロジスティック回帰分析を行ったところ、LW 値のオッズ比は 1.150 (95%信頼区間

1.040-1.28：P<0.05）であり、LW 値は胎児心拍の有無との関連が認められた（表 3)。

【考察】

本研究では LW を用いた胚評価の有用性を調査するために、当院で過去に行った胚移植データを用いて検討を行った。その結果、検討 1 では LW 値を 4 群に分けた際に LW 値が最も低い群 (poor）と最も高い群（excellent）の間に、胎児心拍陽性率において有意な差が認められた。さらに検討 2 では、胎児心拍の有無に影響を及ぼすと考えられる妻年齢と既往胚移植回数を交絡因子として補正し、ロジスティック回帰分析を行ったところ LW 値は胎児心拍の有無に影響を及ぼすことが明らかとなった。今回の結果から、移植する胚盤胞を選択する際の評価方法として LW を用いることは臨床上有用性があることが示唆された。また、今回の研究に利用した胚を Gardner 分類で評価した場合、最良グレード（4AA、5AA、6AA）の胚の胎児心拍陽性率は 55.4% (41/74、未発表データ）であり、Excellent 群の 64.3%よりも低い傾向が見られ、LW は Gardner 分類による評価よりも妊孕性の高い胚を選択できる可能性が考えられた。

Gardner 分類による胚評価では、胚盤胞腔の拡がりや孵化の状態を指標に 6 ステージに分け、さらにステージ 3 以降は内細胞塊と栄養外胚葉をそれぞれ 3 段階で評価する。その評価の境界はあいまいで評価者の主観に委ねられているため、評価者間で評価に差異が生じることがある[5]。評価者間の胚評価の差異を最小限にするために、一人の評価者が全ての胚評価を行ったり、複数の評価者で意見を一致させること[6]や、評価を単純化して評価パターンを減らす[7]ことが有効であると報告されているが、それでも評価の統一は難しいと言える。これに加え、Gardner 分類の胚評価パターンは全部で 38 通りあり、一見どの胚が妊娠の期待の高い胚か分かりにくいという欠点もある。一方の LW は、評価が客観的であり、同じ画像を何度評価させても同じ結果が返ってくるため再現性も高い。さらに、LW の評価結果は 0 から 10 の量的データであることから、どの胚が妊娠の期待が高い胚なのか説明と理解が容易になり、医師と患者間での移植胚の選択もスムーズになると考えられる。培養室内においても、胚評価に要する時間が短縮されることで胚をインキュベーター外に出す時間も短縮され、培養の質の向上が期待される。さらには胚評価に関するトレーニングの短縮や、評価者間の評価の差異から生じるトラブルの解消も期待される。しか

し一方で、同一胚でも焦点を変えて撮影した画像では LW 値が異なる点（図 1）が LW の欠点と言える。開発元は内細胞塊に焦点を合わせた画像を胚評価に用いることを推奨しており、撮影の際には注意が必要である。

人工知能を活用した胚評価として LW の他に、iDA Score (Vitrolife) がある。iDA Score はタイムラプスインキュベーターに搭載された胚評価システムであり、培養中の胚の形態や発育動態を自動的に解析し点数化する [8]。iDA Score に基づく高グレード胚は低グレード胚と比べて流産率が低下し、生児獲得率が上昇するとの有用性が報告されている [9]。iDA Score は培養中の発育動態も加味して詳細に評価している点が LW との違いであるが、高価なタイムラプスインキュベーターを導入しなければならない点や、常にタイムラプスインキュベーターで培養しないと評価ができない点が難点である。一方の LW は胚盤胞の画像を 1 枚用意すれば評価が可能であるため利便性が高い。

ヨーロッパ生殖医学会は 2017 年に ART の成績に関する重要業績評価指標（KPI）を制定した [10] が、培養室ではこういった指標と自施設の成績を比較しながら培養環境を見直し、向上させていかなければならない。また、論文や学会発表等で「良好胚盤胞」を指標として成績を比較しているものを見かけるが、「良好」とする胚の基準や判定が各施設や評価者に委ねられており、データの客観性や共通性に欠ける。このような中で、LW のような客観的な胚評価システムは、各施設で共通の指標を設定するためにも役立つと考える。例えば、LW 値が 9.0 以上の胚を高グレード胚としてその発生割合を比較したり、高グレード胚を移植した時の妊娠率を KPI として制定したりするといった活用方法が考えられる。今回の検討では、LW 値 9.0 以上の excellent 群の胎児心拍陽性率は 64.3%であったが、Diakiw らの報告 [3] では同様の群の胎児心拍陽性率は 64.6%であり、ほぼ同じ成績であった。このことから、Diakiw らの施設と我々の施設は胎児心拍陽性率に関してほぼ同水準のクオリティを有している施設ということができると考えられる。

今回の結果から、LW を用いることで胚移植後の胎児心拍が陽性となる可能性が高い胚を選択することが可能であり、人工知能に基づいた LW による胚評価の臨床上の有用性が明らかとなった。これにより、客観性が高い胚評価が可能となり、さらには、患者の理解度の向上や培養の質の向上も期待される。しかしながら、現時点では培養 5 日目の胚盤胞でしか評価が行えず、培養

6 日目の胚盤胞は対象外となっている点は改善点である。今後、LW の胚評価システムとしての精度向上や解析パターンの拡大に期待するとともに、ユーザー側としては妻年齢や胚培養時間と LW 値の関連性などを細かく検討していく必要がある。

【利益相反】

開示すべき利益相反は無い。

【参考文献】

1. Gardner DK, Lane M, Stevens J, Schlenker T, Schoolcraft WB. Blastocyst score affects implantation and pregnancy outcome: towards a single blastocyst transfer. Fertil Steril 2000; 73: 1155-8.
2. Zhao YY, Yu Y, Zhang XW. Overall Blastocyst Quality, Trophectoderm Grade, and Inner Cell Mass Grade Predict Pregnancy Outcome in Euploid Blastocyst Transfer Cycles. Chin Med J 2018; 131: 1261-7.
3. Diakiw SM, Hall JMM, VerMilyea M, et al. An artificial intelligence model correlated with morphological and genetic features of blastocyst quality improves ranking of viable embryos. Reprod Biomed Online 2022; 45: 1105-17.
4. Kuwayama M, Vajta G, Kato O, Leibo SP. Highly efficient vitrification method for cryopreservation of human oocytes. Reprod Biomed Online 2005; 11: 300-8.
5. Storr A, Venetis CA, Cooke S, Kilani S, Ledger W. Inter-observer and intra-observer agreement between embryologists during selection of a single Day 5 embryo for transfer: a multicenter study. Hum Reprod 2017; 32: 307-14.
6. Baxter Bendus AE, Mayer JF, Shipley SK, Catherino WH. Interobserver and intraobserver variation in day 3 embryo grading. Fertil Steril 2006; 86: 1608-15.
7. Richardson A, Brearley S, Ahitan S, Chamberlain S, Davey T, Zujovic L, Hopkisson J, Campbell B, Raine-Fenning N. A clinically useful simplified blastocyst grading system. Reprod Biomed Online 2015; 31: 523-30.
8. Berntsen J, Rimestad J, Lassen JT, Tran D, Kragh MF. Robust and generalizable embryo selection based on artificial intelligence and time-lapse image sequences. PLoS One 2022; 17: e0262661.
9. Ueno S, Berntsen J, Ito M, Okimura T, Kato K. Correlation between an annotation-free embryo scoring system based on deep learning and live birth/neonatal outcomes after single vitrified-warmed blastocyst transfer: a single-centre, large-cohort retrospective

study. J Assist Reprod Genet 2022; 39: 2089-99.

10. ESHRE Special Interest Group of Embryology and Alpha Scientists in Reproductive Medicine. The Vienna consensus: report of an expert meeting on the development of ART laboratory performance indicators. Reprod Biomed Online 2017; 35: 494-510.

Table. 1　各群の患者背景

Group	Poor	Fair	Good	Excellent	Total
Range of LW score	0.0 - 2.4	2.5 - 7.4	7.5 - 8.9	9.0 - 10.0	
No. cycles	29	82	31	56	198
Age mean±SD	37.1 ± 4.2	36.2 ± 4.6	34.3 ± 4.5	35.1 ± 4.8	35.7 ± 4.6
Median (IQR) number of transferred	1 (0 - 1)	0.5 (0 - 2)	1 (0 - 1)	0 (0 - 1)	0 (0 - 1)
Causes of infertility					
Tubal factor	0 (0%)	6 (7.3%)	1 (3.2%)	3 (5.4%)	10 (5.1%)
Endometriosis	3 (10.3%)	8 (9.8%)	1 (3.2%)	3 (5.4%)	15 (7.6%)
Male factor	9 (31.0%)	17 (20.7%)	5 (16.1%)	8 (14.3%)	39 (19.7%)
Ovulation	1 (3.4%)	3 (3.7%)	5 (16.1%)	4 (7.1%)	13 (6.6%)
Low Anti-Mullerian Hormone	1 (3.4%)	1 (1.2%)	0 (0%)	0 (0%)	2 (1.0%)
Age factor	2 (6.9%)	4 (4.9%)	3 (9.7%)	7 (12.5%)	16 (8.1%)
Multiple	5 (17.2%)	6 (7.3%)	3 (9.7%)	5 (8.9%)	19 (9.6%)
Unknown	8 (27.6%)	37 (45.1%)	13 (41.9%)	26 (46.4%)	84 (42.4%)

Table. 2　LW 値で 4 群に分けた際の胎児心拍陽性率の比較

Groups	Poor	Fair	Good	Excellent
No. cycles	29	82	31	56
No. (%) of positive fetal heart beat	8 (27.6%) [a]	35 (42.7%)	13 (41.9%)	36 (64.3%) [b]

[a,b] 異符号間に有意差あり（$P < 0.05$）

Table. 3　LW 値が胎児心拍の有無に及ぼす影響

	Odd rate	95% confidence interval	P value
LW score	1.150	1.040 – 1.28	0.009
Age	0.915	0.855 – 0.98	0.012
No. of transferred	0.831	0.630 – 1.10	0.189

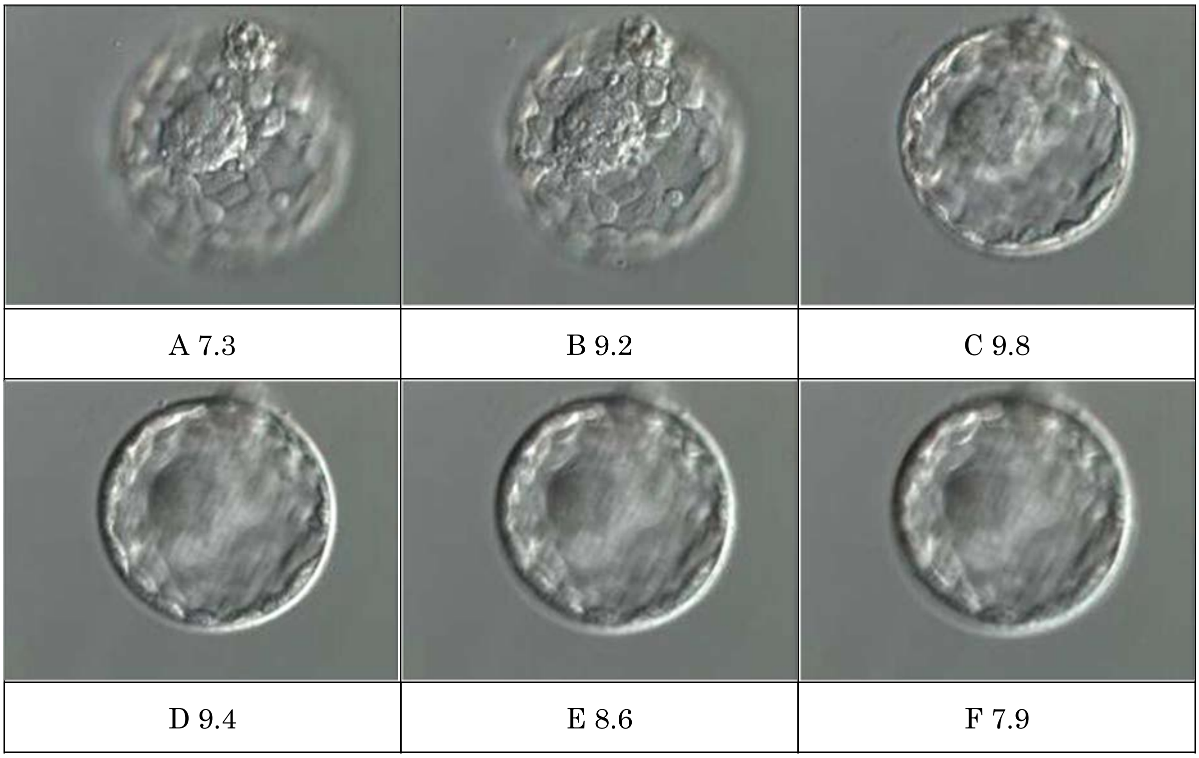

Figure 1　同一胚を焦点を変えて撮影した際の LW 値の変化

A から F に徐々に焦点を変えて撮影した。胚直径は 180μm。
